# Role of tumor-derived exosomes and immune cells in osteosarcoma progression and targeted therapy

**DOI:** 10.3389/fimmu.2025.1658358

**Published:** 2025-11-26

**Authors:** Jingchao Wang, Kuohao Shi

**Affiliations:** Translational Medicine Center, Honghui Hospital, Xi’an Jiaotong University, Xi’an, China

**Keywords:** osteosarcoma, tumor-associated macrophages, exosome, immunosuppression, immune checkpoint inhibitors, immunotherapy

## Abstract

Osteosarcoma, the most common primary malignant bone tumor, poses significant clinical challenges due to its aggressive nature, high metastatic potential, and resistance to conventional therapies. Despite improvements in surgical and chemotherapeutic approaches, survival rates for relapsed or metastatic disease remain poor. Recent advances in understanding the tumor immune microenvironment (TIME) and exosome biology have uncovered critical mechanisms driving osteosarcoma progression, immune evasion, and therapeutic resistance. Tumor-associated macrophages (TAMs), particularly the M2 phenotype, dominate the osteosarcoma immune landscape and contribute to immunosuppression through cytokine secretion and modulation of T cell function. Exosomes, as intercellular messengers, further exacerbate tumor progression by transporting oncogenic proteins, immunosuppressive factors (TGF-β), miRNAs, and drug-resistance molecules. These vesicles also influence critical signaling cascades including Wnt/β-catenin and TGF-β pathways, shaping both local and systemic tumor responses. This review delineates the roles of immune cells and tumor-derived exosomes in osteosarcoma biology and evaluates emerging immunotherapeutic strategies, including immune checkpoint inhibitors, CAR-T cells, tumor vaccines, cytokine-targeted agents, and combination therapies. We highlight ongoing clinical trials, numerical efficacy metrics, and the translational promise of exosome-based diagnostics and therapeutics. Ultimately, integrated approaches targeting both the TIME and exosome-mediated mechanisms may yield more effective and durable treatments for osteosarcoma patients.

## 1 Introduction

Osteosarcoma is the most common primary malignant bone tumor, accounting for approximately 20% of all primary malignant bone tumors worldwide (1). It predominantly affects children, adolescents, and elderly individuals. Traditional amputation yields a 5-year survival rate of <20% (2), but advancements in surgery, radiotherapy (RT), chemotherapy (CT), targeted therapy, and multidisciplinary approaches have improved survival to 60%–80% (2). Despite this, osteosarcoma retains the highest mortality among bone malignancies (3), with metastatic/recurrent cases showing <30% long-term survival (4), necessitating more effective therapies.

Recent insights into the tumor immune microenvironment (TIME) have spurred progress in immunotherapy, including tumor vaccines, cytokine targeting, immune checkpoint inhibition, adoptive cell therapy, and combinatorial strategies (5, 6). However, their efficacy in osteosarcoma requires further exploration. Concurrently, exosome research has revealed their role in intercellular communication, transporting proteins, signaling factors, and miRNAs to modulate recipient cell function (7). Tumor-derived exosomes also contribute to drug resistance by expelling anticancer agents (8). Exosome analysis aids in early diagnosis, treatment guidance, and prognosis assessment, while engineered exosomes serve as potential drug/gene delivery vehicles. This review focuses on the immune microenvironment of osteosarcoma, discussing the immune therapeutic strategies associated with it, the translational applications of these strategies in osteosarcoma treatment, and evaluating the potential challenges and opportunities. We also look forward to future research directions in this field.

## 2 Immune microenvironment and exosome in osteosarcoma

### 2.1 The immune microenvironment of osteosarcoma

TIME in osteosarcoma is a complex system consisting of tumor cells, immune cells, fibroblasts, endothelial cells, and extracellular components. These elements interact through cytokines, chemokines, and growth factors (9). In osteosarcoma tissues, monocytes and macrophages represent 70-80% of bone marrow cells, with dendritic cells (DCs) making up less than 5% (10). Tumor-associated macrophages (TAMs) are the dominant immune cell type, constituting over 50% of the immune cell population (11, 12). TAMs are classified into M1 and M2 types. M1 TAMs exhibit pro-inflammatory and anti-tumor functions and are typically associated with improved prognosis (13). In contrast, M2 TAMs, induced primarily by IL-4 and IL-13 through activation of the STAT6 pathway, adopt an immunosuppressive phenotype characterized by the secretion of anti-inflammatory cytokines and growth factors (14). These M2-polarized TAMs play pivotal roles in osteosarcoma progression by secreting vascular endothelial growth factor (VEGF), which promotes neovascularization and tumor perfusion, and transforming growth factor-β (TGF-β), which contributes to extracellular matrix deposition and tumor-associated fibrosis. Furthermore, they suppress cytotoxic T lymphocyte function and facilitate osteosarcoma cell invasion and metastasis (15). The polarization of M1 to M2 TAMs plays a significant role in osteosarcoma progression. M2 TAMs induce high mobility group box 1 (HMGB1) expression in osteosarcoma cells, promoting migration and invasion, while HMGB1 further enhances M2 polarization (16). In addition, tumor-derived exosomes containing TGF-β2 can induce M2 polarization, thereby sustaining an immune-suppressive environment (17). Reversing M2 TAMs to M1 has emerged as a promising therapeutic approach (18). Additionally, cancer stem cells (CSCs) interact with TAMs to maintain osteosarcoma development (19), macrophage phagocytic activity modulates their polarization through the P38/STAT3 axis, promoting PD-L1 expression and enhancing immune evasion (20).

### 2.2 The role of exosomes in the tumor microenvironment of osteosarcoma

**Figure 1:**
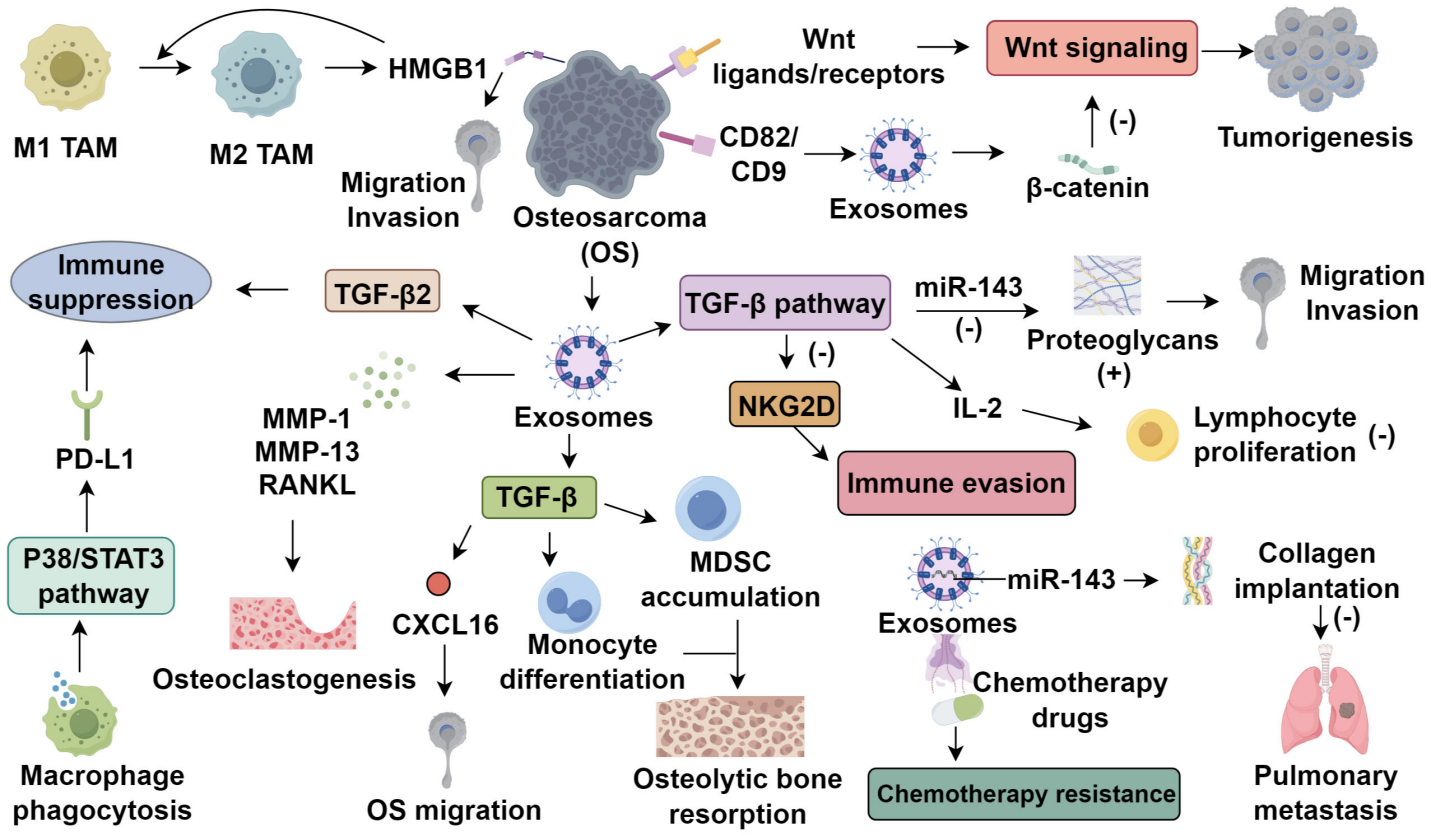
The role of tumor-derived exosomes in the tumor microenvironment of osteosarcoma.

Exosomes, 40–100 nm vesicles released upon multivesicular body fusion with the plasma membrane, carry proteins, double-stranded DNA, and various RNAs (21). Their biogenesis is governed by Rab GTPases (Rab27a/b), ceramide metabolism, and tumor-associated pH changes (22, 23), and their membrane is enriched with scaffolding proteins (tubulin, annexins, Alix, TSG101), tetraspanins (CD9, CD63), and cell-specific markers such as MHC-I (24, 25). They encapsulate signaling molecules involved in oncogenic cascades, including Wnt/β-catenin and TGF-β pathways (26). Tumor microenvironmental stressors, notably hypoxia and acidosis, significantly enhance exosome release (27). In osteosarcoma, hypoxia-induced HIF-1α upregulates Rab22A, promoting multivesicular body formation and enriching exosomes with pro-angiogenic and immunosuppressive factors, thereby facilitating metastasis (27–29). Tumor-derived exosomes critically shape the TME, enhancing angiogenesis, metastasis, and immune evasion (30–32). Garimella et al. (33) characterized osteosarcoma-derived exosomes in a bioluminescent murine model using transmission electron microscopy. These exosomes contain matrix metalloproteinases (MMP-1, MMP-13) and RANKL, contributing to matrix degradation, osteoclastogenesis, and potential prognostic value (34). Exosomal TGF-β modulates CXCL16 in osteoclasts and promotes monocyte-to-MDSC differentiation, exacerbating osteolytic disease (35, 36). CD9 modulates osteoclast differentiation and metastatic tropism, with blockade by KMC8 inhibiting osteoclastogenesis (37, 38). Additionally, intracellular mediators such as calcium, cAMP, and P2X7 receptor activation regulate exosome secretion (39–41). Therapeutic strategies targeting exosome biogenesis or function, such as Rab27a knockdown, CD9 blockade, or RANKL neutralization, may disrupt osteolytic signaling and preserve bone integrity in osteosarcoma (**Figure 1**).

## 3 The role of exosome-mediated signaling pathways in osteosarcoma

### 3.1 Wnt signaling pathway

Targeted therapy of the Wnt pathway in tumors has drawn significant interest. Chen et al. (42) found that osteosarcoma cell lines expressing Wnt ligands/receptors exhibit differentiation potential via autocrine Wnt signaling. Kansara et al. (43) reported that Wnt inhibitory factor 1 knockout increased osteosarcoma incidence in mice, suggesting Wnt activation promotes tumorigenesis. Conversely, Cleton-Jansen et al. (44) observed Wnt downregulation in osteosarcoma specimens versus osteoblastomas and mesenchymal stem cells. Studies also noted elevated Dkk-1 expression at tumor margins, correlating with invasiveness; Dkk-1 blockade inhibited osteosarcoma cell proliferation via activation of the WNT/β-catenin pathway (45). Besides, osteoblast-derived exosomes effectively inhibited the proliferation of osteosarcoma cells and promoted their mineralization via RG4/Wnt signaling pathway (46). These seemingly contradictory findings, Wnt pathway activation associated with tumor progression in some studies and suppression in others, highlight the context-dependent nature of Wnt signaling in osteosarcoma. Several factors may account for this dichotomy, including tumor heterogeneity, differences between early and late disease stages, and the interplay between canonical (β-catenin-dependent) and non-canonical Wnt pathways (44, 47). These findings not only emphasize the dualistic nature of Wnt signaling but also underscore the key role of exosomes in modulating and distributing Wnt pathway activity within the osteosarcoma microenvironment, informing future targeted therapies.

### 3.2 TGF-β signaling pathway

TGF-β regulates cell proliferation, differentiation, extracellular matrix production, angiogenesis, apoptosis, and immune responses (48). Li et al. (49) demonstrated in 60 osteosarcoma specimens that TGF-β1 upregulates proteoglycans by suppressing miR-143, enhancing tumor invasiveness and metastasis. *In vitro* studies have revealed that TGF-β1 enhances the migratory capacity of various osteosarcoma cell lines, a phenomenon likely linked to its ability to induce epithelial–mesenchymal transition (EMT)-like changes. Moreover, TGF-β1 has been implicated in promoting angiogenesis within the osteosarcoma microenvironment (48). Exosomal TGF-β1 suppresses IL-2-induced lymphocyte proliferation, exhibiting 1400-fold greater immunosuppression than soluble TGF-β1 (50, 51). Tumor−derived exosomal TGF−β1 engages TGF−β receptor II on NK and CD8^+^ T cells, activating the SMAD2/3 pathway and leading to transcriptional repression of NKG2D, a critical activating receptor required for cytolytic granule release. This downregulation diminishes immune recognition and killing of osteosarcoma cells, allowing tumors to escape immune surveillance (52–54). Kawano et al. (55) found anti-TGF-β antibodies inhibit osteosarcoma proliferation and stimulate systemic immunity, suggesting therapeutic potential. Beyond their immunosuppressive effects, exosomal TGF-β1 particles represent promising delivery vehicles for targeted immunomodulation. Engineering exosomes to either block or reverse TGF-β1-mediated immunosuppression—such as loading antagonists or small interfering RNAs against TGF-β signaling—may offer a novel therapeutic avenue in overcoming immune evasion and restoring anti-tumor immunity in osteosarcoma (56).

### 3.3 Exosome-mediated miRNAs

Emerging evidence highlights the pivotal role of exosomal miRNAs in osteosarcoma progression and immune regulation. miR-143 suppresses pulmonary metastases in murine osteosarcoma models (57), promoting apoptosis and reducing tumor growth upon upregulation (58). Exosomal packaging enhances the stability and intercellular transport of miRNAs, enabling them to function as gene transfer agents. Shimbo et al. (59) showed that synthetic miR-143, delivered via exosomes from bone marrow mesenchymal stem cells, significantly reduced OS metastatic potential, with greater efficacy than conventional transfection. Other exosomal miRNAs further contribute to OS pathogenesis (60). Exosomal miR-21, miR-21, commonly overexpressed in OS, promotes proliferation, metastasis, drug resistance, and immune evasion via PTEN suppression and PI3K/AKT signaling (61, 62). Similarly, exosomal miR-148a enhances tumor progression by targeting PTEN and DNMT1 (63, 64). These findings underscore the diverse and critical functions of exosome-associated miRNAs in shaping the TIME and facilitating osteosarcoma pathogenesis. Exosome membranes harbor tumor-associated antigens, MHC molecules, and co-stimulatory signals, capable of eliciting cytotoxic T cell responses (65, 66). OS cells often downregulate MHC class molecules, impairing antigen presentation. Tumor-derived membranous vesicles (Texo) isolated from osteosarcoma cells can sensitize DCs, activating T cells to suppress tumor growth (59). These insights support the therapeutic potential of exosome-based strategies in osteosarcoma immunotherapy.

### 3.4 Exosomes and tumor drug resistance

The resistance of tumor cells to docetaxel is positively correlated with the amount of exosome secretion (8). Shedden et al. (67) observed that exosomes encapsulate and expel a variety of anti-cancer drugs, a phenomenon observed in multiple tumors, which may be related to the sensitivity of the tumors to anti-cancer drugs. Chemotherapeutic drugs can be expelled from tumor cells via exosomes, while antibody-based targeted drugs can also be neutralized. In addition to passive drug sequestration and efflux, exosomes actively contribute to chemoresistance by transporting drug-resistance-related proteins and regulatory RNAs. Notably, exosomes derived from chemoresistant osteosarcoma cells have been shown to carry functional P-glycoprotein (P-gp/ABCB1), a membrane-associated ATP-binding cassette (ABC) transporter (68, 69). Upon transfer to sensitive cells, exosomal P-gp can confer multidrug resistance by enhancing drug efflux capacity, thereby reducing intracellular drug accumulation and therapeutic efficacy. Moreover, these exosomes derived from chemoresistant osteosarcoma cells facilitate the development of drug resistance by delivering miR-331-3p and activating autophagy pathways. Targeted suppression of miR-331-3p expression may represent a potential strategy to overcome chemoresistance in osteosarcoma (70). Chemotherapy plays an important role in the treatment of osteosarcoma. Therefore, exploring the specific mechanisms by which osteosarcoma cell exosomes encapsulate, transport, and excrete chemotherapy drugs and their metabolic products is crucial for improving the efficacy of chemotherapy in osteosarcoma patients. Targeting exosome biogenesis or blocking the uptake of drug-resistant exosomes represents a promising strategy to overcome chemotherapy resistance in osteosarcoma.

## 4 Immunotherapy for osteosarcoma

### 4.1 Immune checkpoint inhibition

T cell activation, crucial for antitumor immunity, relies on TCR-MHC and CD28-CD80/CD86 co-stimulation (71). Immune checkpoints such as PD-1 and CTLA-4 inhibit these pathways by by engaging PD-L1 or B7 ligands, leading to immune evasion and establishing the rationale for immune checkpoint inhibitors (ICIs). PD-1 modulates autoimmunity, while PD-L1, expressed on tumor cells, inversely correlates with prognosis (72). Osteosarcoma studies show elevated PD-L1 serum levels, often exceeding PD-1, with high PD-L1 linked to recurrence/metastasis (73, 74), suggesting its regulation as a therapeutic target. ICIs act by blocking checkpoint proteins, releasing immune suppression and activating tumor cell killing. In osteosarcoma models, PD-1 inhibition promotes M1/M2 macrophage polarization in lung metastases, inducing regression (75). Anti-PD-L1 antibodies enhance NK cell cytotoxicity against osteosarcoma (76), suggesting PD-1/PD-L1 blockade may benefit patients. Clinical trials show PD-L1 expression/amplification correlates with ICI response, though optimal thresholds remain undefined (77, 78). Avelumab (anti-PD-L1) is under evaluation in a Phase II trial (NCT03006848), while apatinib plus camrelizumab improved PFS in PD-L1-positive advanced osteosarcoma (79). Another PD-1-targeting trial (NCT04359550) demonstrates socazolimab’s efficacy in postoperative maintenance. CTLA-4, a CD28 homolog, binds B7 ligands, enabling immune escape (80), observed in osteosarcoma. Anti-CTLA-4 enhances CTL activity (81), supporting CTLA-4 inhibition as a potential therapeutic strategy. However, ICI efficacy is inconsistent in osteosarcoma, likely due to its low tumor mutational burden (TMB) and heterogeneous PD-L1 expression, limiting neoantigen availability and immune recognition (82, 83). Notably, tumor-derived exosomes carrying membrane-bound PD-L1 exert systemic immunosuppressive effects by engaging PD-1 on T cells independent of direct tumor–T cell contact, contributing to therapy resistance and immune evasion in various cancers, including osteosarcoma (84). This mechanism suggests that PD-L1–positive exosomes may serve as both a biomarker for ICI resistance and a potential therapeutic target in osteosarcoma.

### 4.2 Adoptive cell therapy

Adoptive cell therapy (ACT), particularly chimeric antigen receptor T cell (CAR-T) therapy, genetically engineers autologous T cells to recognize tumor-specific antigens, thereby inducing robust antitumor responses (85). While effective in hematological malignancies, CAR-T therapy faces challenges in solid tumors like osteosarcoma due to antigen heterogeneity, limited persistence, and the immunosuppressive TME (86). GD2, frequently overexpressed in osteosarcoma, has emerged as a key target. GD2-directed CAR-T cells demonstrate potent cytotoxicity, with Phase I trials confirming safety and feasibility (87, 88). However, PD-L1/PD-1–mediated immunosuppression and intratumoral variability in GD2 expression contribute to immune evasion and antigen escape (89). Moreover, dynamic downregulation of GD2 following immune pressure further reduces CAR-T efficacy, emphasizing the need for multi-targeting strategies (90). The TME, enriched in M2-like tumor-associated macrophages (TAMs), regulatory T cells (Tregs), and myeloid-derived suppressor cells (MDSCs), releases suppressive cytokines (TGF-β, IL-10) and depletes essential nutrients (arginine, tryptophan), collectively impairing CAR-T function (91, 92). Overcoming these barriers may require combinatorial approaches involving checkpoint blockade, cytokine modulation, or metabolic reprogramming. Additionally, CAR-T cell trafficking is hindered by a dense extracellular matrix, abnormal vasculature, and lack of chemokine gradients (91). To improve tumor infiltration, strategies include engineering CAR-T cells to express chemokine receptors such as CCR2 (responsive to CCL2), or combining therapy with matrix-degrading enzymes or low-dose radiotherapy (91). Beyond GD2, emerging targets include alkaline phosphatase 1 (ALP-1), specific to metastatic osteosarcoma, and interleukin-11 receptor α (IL-11Rα), a bone-associated antigen. Preclinical data suggest ALP-1- and IL-11Rα-targeted CAR-T cells offer enhanced tumor specificity and cytotoxicity (89, 93, 94). Despite promise, further research is needed to optimize CAR-T efficacy in osteosarcoma.

### 4.3 Tumor vaccines

Tumor vaccines are an emerging immunotherapeutic strategy that harness tumor-associated antigens to elicit specific immune responses capable of targeting malignant cells. Among these, dendritic cell (DC) vaccines have shown the most clinical promise in osteosarcoma (95). As professional antigen-presenting cells, DCs prime cytotoxic T lymphocytes (CTLs), amplifying antitumor immunity. DC vaccines can be derived autologously, from patient-derived blood or bone marrow, or allogeneically, using donor-derived DCs. In both cases, DCs are pulsed with tumor antigens ex vivo prior to reinfusion. Although most DC-based vaccines remain in early-phase clinical trials, the advancement of agents such as DCVax-L to Phase III underscores their therapeutic potential (96). However, DC vaccine efficacy in osteosarcoma remains modest, prompting investigation into combinatorial regimens. Notably, irreversible electroporation (IRE) has been shown to enhance antigen release and immunogenicity, thereby augmenting DC vaccine efficacy and systemic antitumor responses (97). In parallel, peptide vaccines—constructed from tumor-associated or neoantigen-derived epitopes—offer a more defined and potentially personalized immunotherapeutic platform. Neoantigen-based approaches are particularly promising due to their high immunogenicity and reduced risk of central tolerance, and are now being explored in sarcomas (98, 99). Furthermore, exosome-loaded vaccines have emerged as a novel strategy to enhance antigen presentation and immune priming, making them attractive candidates for future osteosarcoma vaccine development (100).

### 4.4 Targeting cytokines

Vascular endothelial growth factor (VEGF) is a pivotal cytokine regulating endothelial cell growth, differentiation, and angiogenesis, promoting tumor progression by supplying oxygen and nutrients. Despite clinical trials of VEGF-targeting drugs (regorafenib, cabozantinib, sorafenib, pazopanib, bevacizumab), most show no significant median survival time (MST) improvement, indicating limited efficacy (101). However, some demonstrate therapeutic potential, such as regorafenib, currently under evaluation in trials (NCT02389244, EUCTR2013-003910-42, EUCTR2019-002629-31, NCT04698785, NCT04803877). DUFFAUD et al. (102) reported regorafenib significantly prolonged progression-free survival (PFS) in advanced osteosarcoma, supported by subsequent studies (103, 104). Apatinib, a selective VEGFR2 inhibitor, suppresses Sox2 via STAT3, reducing doxorubicin resistance (105), and induces apoptosis by inhibiting VEGFR2/STAT3/BCL-2 signaling (106). Ongoing trials (NCT06125171, NCT05277480, ChiCTR2200062550) and phase II data support its use in chemotherapy-resistant advanced osteosarcoma (107). Similarly, surufatinib shows promise in a trial (NCT05106777) for chemotherapy-refractory cases, with ongoing recruitment (108). Beyond pharmacotherapy, VEGFR2 gene silencing offers a novel strategy for pulmonary metastasis. YU et al. (109) proposed a genetic circuit delivering VEGFR2 siRNA via plasmid DNA, enabling targeted lung delivery for metastatic osteosarcoma treatment. In addition to soluble VEGF, tumor-derived exosomes have been shown to encapsulate and transport VEGF molecules, thereby facilitating localized angiogenic signaling in a protected vesicular form. These VEGF-positive exosomes can be internalized by endothelial cells in the tumor microenvironment, enhancing vascular proliferation and remodeling (110). In osteosarcoma, such exosomal delivery may contribute to aberrant neovascularization and metastatic spread, highlighting exosomal VEGF as a novel target for anti-angiogenic therapies.

### 4.5 Combination immunotherapy

Combination immunotherapy for osteosarcoma has garnered significant attention, with studies highlighting synergistic effects of dual checkpoint inhibition. LUSSIER et al. (111) demonstrated that anti-PD-L1 antibodies reduced PD-1 expression on CD8^+^ T cells while increasing CTLA-4 in tumor-infiltrating lymphocytes (TILs), suggesting complementary roles in suppressing CTL responses. Metastatic osteosarcoma mouse model showed complete tumor control and prevented immune escape in 50% of cases with PD-L1/CTLA-4 blockade. D’ANGELO et al. (112) reported 49% survival with nivolumab/ipilimumab versus 32% with nivolumab alone in metastatic sarcoma. Combining ICIs with other modalities enhances efficacy. Anti-CTLA-4 antibodies paired with dendritic cell vaccines improved responses in osteosarcoma models (113), while ICIs augmented CAR-T cell activity by reversing T cell exhaustion (114). Targeted combinations also show promise. GD2 antibodies with IL-2/GM-CSF boosted solid tumor responses (115, 116), while GD2 plus cisplatin induced ER-mediated apoptosis in osteosarcoma (117). Low-dose doxorubicin enhanced dendritic cell efficacy via immunogenic cell death (118). These findings underscore combination therapy’s potential to improve response rates and durability, particularly for treatment-resistant cases. As mechanistic understanding advances, multimodal approaches may redefine osteosarcoma management (**Table 1**).

**Table 1:** Exosome-mediated mechanisms and therapeutic applications in osteosarcoma.

Aspect	Key components	Mechanisms	Implications in Osteosarcoma	Therapeutic strategies
Tumor-derived Exosomes	Proteins, miRNAs (miR-143), TGF-β, Wnt ligands	Promote immune evasion, metastasis, and drug resistance	Induce M2 TAM polarization, suppress T cells, enhance osteoclastogenesis	Exosome inhibition; exosome engineering for miRNA delivery
TAM Polarization	M1 (anti-tumor), M2 (pro-tumor)	M2 TAMs secrete IL-10, TGF-β; support tumor growth	Abundant in TIME; facilitate immune suppression and metastasis	Reprogramming M2 to M1 via cytokine modulation or miRNA intervention
Signaling Pathways	Wnt/β-catenin, TGF-β, P38/STAT3	Alter gene expression, promote proliferation/invasion, inhibit immune response	Drive tumor progression and immunosuppression via exosomal signaling	Targeted inhibitors of Wnt or TGF-β pathways
Immune Checkpoints	PD-1/PD-L1, CTLA-4	Inhibit T cell activation	High PD-L1 in recurrent/metastatic OS; immune escape	ICIs: nivolumab, ipilimumab, camrelizumab
Combined Immunotherapy	CAR-T, vaccines, cytokine inhibitors	Synergize immune response, overcome resistance	Improve outcomes in refractory/metastatic osteosarcoma	CAR-T + ICI; DC vaccine + IRE; VEGFR inhibitors + ICIs

## 5 Conclusion

Osteosarcoma’s complex TIME and exosome-mediated interactions present both challenges and opportunities for therapeutic innovation. The immunosuppressive milieu, driven by M2-polarized TAMs and exosome-facilitated signaling, underscores the need for strategies that reprogram the TIME while targeting tumor-intrinsic pathways. Immunotherapy, particularly ICIs and CAR-T cells, has shown encouraging but variable efficacy, necessitating biomarker-driven patient stratification and combinatorial approaches to enhance durability. Exosomes, as mediators of metastasis, drug resistance, and intercellular communication, offer dual utility as diagnostic biomarkers and therapeutic vehicles, especially when engineered for targeted drug or miRNA delivery.

Key challenges include overcoming antigen heterogeneity, mitigating immune evasion, and optimizing exosome-based delivery systems. Future research should prioritize elucidating mechanisms of exosome-mediated immune suppression, developing exosome-mimetic nanotherapies, refining CAR-T designs for solid tumors, and integrating immunotherapy with targeted agents or chemotherapy to exploit immunogenic cell death. Clinical translation will depend on robust preclinical models and adaptive trial designs to evaluate emerging combinations. By bridging insights from TIME biology and exosome science, this field holds transformative potential to improve outcomes for osteosarcoma patients.
